# Socio-demographic characteristics, clinical profile and prevalence of existing mental illness among suicide attempters attending emergency services at two hospitals in Hawassa city, South Ethiopia: a cross-sectional study

**DOI:** 10.1186/s13033-017-0136-4

**Published:** 2017-04-20

**Authors:** Moges Ayehu, Tarekegn Solomon, Kinfe Lemma

**Affiliations:** 1 School of Medicine, Hawassa University, Hawassa, Ethiopia; 2 School of Public and Environmental Health, Hawassa University, Hawassa, Ethiopia; 3 College of Medicine and Health Sciences, Hawassa University, Hawassa, Ethiopia

**Keywords:** Suicide, Suicidal attempt, Psychiatric disorders, Mental illness, Impulsivity, Low and middle income countries, Ethiopia

## Abstract

**Background:**

Suicide is a major public health problem worldwide. It contributes for more than one million deaths each year. Since previous suicidal attempt was considered as the best predictor of future suicide, identifying factors behind suicidal attempt are helpful to design suicide prevention strategies. The aim of this study was to assess socio-demographic characteristics, clinical profile and prevalence of existing mental illness among patients presenting with suicidal attempt to emergency services of general hospitals in South Ethiopia.

**Methods:**

We conducted a cross-sectional study on patients presenting with complications of suicidal attempt to emergency departments of two general hospitals in Hawassa city from November, 2014 to August, 2015. Data was collected using semi-structured questionnaire which contained socio-demographic and clinical variables. The Mini International Neuropsychiatric Interview version 5 (MINI PLUs) was used to assess the prevalence of existing mental illness among study participants. Data was entered and analyzed using IBM SPSS statistics 21 software package.

**Results:**

A total of 96 individuals were assessed, of whom 56 (58.3%) were females. The mean age of study participants was 21.5 (8.0) years. The majority, 75 (78.1%), of the study participants were aged below 25 years. Ingesting pesticide poisons and corrosive agent were used by the majority to attempt suicide. Mental illness was found in only three (3.1%) of the study participants. Impulsivity (the time between decision to attempt suicide and the actual attempt of less than 5 min) was reported by 30 (31.2%) of the study participants, of whom 18 (60%) were males. Males were found three times more likely to attempt suicide impulsively than women (COR = 3.0, 95% CI 1.2–7.3). Quarreling with family members, facing financial crisis, and having unplanned and unwanted pregnancy were reported by the majority of study participants as immediate reasons to attempt suicide.

**Conclusions:**

The presence of stressful life events and impulsivity behind suicidal behavior of the younger generation implies that designing suicide prevention strategies for this group is crucial. Moreover, further research is needed to systematically examine the relationship between the presence of mental illness and suicidal attempt with a larger sample size and more robust methodology.

## Background

Suicide is a major public health concern worldwide. Over one million suicide deaths, 16 people per 100,000 persons or one death per 40 s each year occur worldwide [1]. Among these deaths 75% of them were reported from low and middle-income countries (LMICs) [2]. According to a World Health Organization report [3], the highest suicide rate which is 44.2 per 100,000 persons per year (70.8 men, 22.1 women) was reported recently from Guyana, a country found in the north eastern edge of South America. North Korea (38.5 per 100,000 persons), South Korea (28.9 per 100,000 persons), Sri Lanka (28.8 per 100,000 persons), and Lithuania (28.2 per 100,000 persons) were the other countries where the highest suicide rates were documented. Among African countries, the highest suicide rates were reported from Mozambique (27.4 per 100,000 persons), Tanzania (24.9 per 100,000 persons), and Burundi (23.1 per 100,000 persons) [3]. In Ethiopia, 7.6 up to 11.5 suicide deaths per 100,000 persons per year were reported [3, 4]. Moreover, suicide is under-reported in various countries due to the high level of stigma, religious and cultural taboos linked to it [5–7]. For example, Vijayakumar reported that the annual estimated suicide rate could be between six and nine times higher than the official figure [8]. Studies reported that before someone committed suicide, depressive thought, thinking that life is not worth living, suicidal thought, suicidal plan and finally suicidal attempt preceded [9–13].

Many studies have shown that the numbers of suicide attempters have been increasing worldwide. According to studies in the past, suicide attempts were 20–40 times more frequent than suicide [14, 15]. On the other hand, attempted suicide was found the leading risk factor for suicide [16–18]. A meta-analysis study has shown that suicide was 30–40 times higher in those individuals who attempted suicide than the general population [19].

The contribution of mental disorders such as bipolar 1 disorder and major depression disorders to attempt suicide was also found significant. For example, an Ethiopian based 10 years population study has shown that 26.3% of patients with major depression and 23.8% of patients with bipolar 1 disorder attempted suicide [20]. In this cohort study, more than one-tenth (13.1%) of patients with schizophrenia also attempted suicide within the study period [20]. According to different studies conducted in the past, the prevalence of mental illness among patients present to the hospital following suicidal attempt was found in the ranges of 60–80% [21–23]. Moreover, socio-demographic factors like young age, female sex, low level of education, history of previous attempt [24, 25], being unemployed and living in urban areas [26, 27] were found highly associated with suicide. Psychosocial factors like problems with love affairs, illegitimate pregnancy, extra-marital affairs and marriage [28], living in socio-economically deprived situations [21], and stressful life events one month prior to suicidal attempt [29] were also found significantly associated with suicidal attempt. In addition to these, many studies in the past had associated suicidal attempt with impulsive behavior; particularly among adolescents [30–33].

Even though having a better understanding of suicidal behavior was very helpful to prevent suicide, there is a paucity of data in developing countries including Ethiopia. Since attempted suicide is very helpful to estimate future suicide, it is good to know factors that are associated with suicidal attempt. Thus, this study was conducted to assess sociodemographic characteristics, clinical profiles and prevalence of existing mental illness among patients presenting with suicidal attempt to emergency services of two general hospitals in South Ethiopia.

## Methods

### Study design and setting

A facility based cross-sectional study was conducted from November 2014 to August 2015 at Hawassa University Referral Hospital and Adare Primary Hospital. Both hospitals are found in Hawassa City, which is located about 270 km south of Addis Ababa. Hawassa University Referral Hospital is the only referral hospital in Southern Nations, Nationalities and Peoples’ Region (SNNPR), and is expected to provide services for more than 18 million people. In contrast, Adare Primary Hospital was established to treat primarily patients coming from Hawassa City. Even though both hospitals have inpatient and outpatient services in major medical disciplines, psychiatric services are limited to outpatient services only. The psychiatric clinic in Hawassa University Referral has two psychiatrists and two psychiatric nurses whereas; Adare Primary Hospital has one psychiatric nurse. Patients with clear cases of mental illness are usually referred directly from the triage to the psychiatric outpatient department (OPD). Moreover, those patients who are referred from the triage to other medical clinics because of poor triaging are referred back to the psychiatric clinic if health professionals working in other disciplines suspect the presence of psychiatric problems in the patient. Patients with suicidal attempts are supposed to be treated at the general emergency OPD and inpatient services till they become medically stable. After having been stabilized medically, they are usually referred to psychiatric outpatient services for psychiatric evaluation and treatment.

### Participants

Participants of this study were all those suicide attempters who consecutively presenting to the emergency and inpatient services of the two general hospitals from November 2014 to August 2015. The emergency and inpatient teams were informed to refer every patient with suicidal attempt to psychiatric OPD after making them medically stable. For the purpose of this study, suicide attempt is defined as “any act of self-damage inflicted with self-destructive intentions” [34, 35]. Patients with accidental injuries without the intention of self-harm, and those who did not survive were excluded from the study. In the study periods one-hundred-one patients were presenting to emergency departments of the two hospitals. Among these, five of them died within few minutes to hours of hospital visit while they were on treatment at emergency OPD. Nine (9.4%) suicide attempters were discharged from emergency OPD since they did not have serious complications which required admission. On the other hand, 87 (86.1%) suicide attempters were admitted to inpatient services either for having serious medical complications or for further observation. After making them medically stable, both the latter groups were interviewed at psychiatric OPD before discharge.

### Assessment

Semi-structured questionnaires were prepared by the investigators containing the following study variables: socio-demographic characteristics, history of previous suicidal attempt, method of suicidal attempt, presence of previously known mental illness, intention of suicidal attempt, immediate reasons for suicide, whether the patient made suicidal attempt impulsively or with premeditative plan, patient condition during arrival, patient outcome after treatment, attitude towards surviving the incident, and presence of known medical illness. Although impulsivity was not defined in a similar way in previous studies, in our study like in the study done by Simon et al. [31], impulsive suicidal attempt was considered to happen if the respondent reported the spending of <5 min between the decision to attempt suicide and the actual attempt. The Mini International Neuropsychiatric Interview version 5 (MINI PLUS) was used to assess the prevalence of an existing mental illness among study participants based on Diagnostic and Statistical Manual-IV edition (DSM-IV) [36]. The instrument was designed to assess major psychiatric disorders such as psychosis, mood disorders, anxiety disorders, suicide, alcohol and drug related disorders, eating disorders, and somatoform disorders. Among childhood disorders, attention deficit hyperactivity disorder (ADHD) and conduct disorders were included, but among personality disorders, only antisocial personality was incorporated in the instrument. MINI PLUS was translated in various languages and its’ validity and reliability was found to be high [34, 35]. Though the instrument was not validated in Ethiopia, it was validated [37–39] and used [40–42] in various African settings. The instrument was also selected based on its broad coverage, easy to use by data collectors and quick administration time. We used the English version of the instrument. Although short time of administration (mean 18.7 + 6 min) was mentioned as one of the advantages of using MINI-Plus [43], in our study the average time required to fill it was 30 min.

### Procedures

Data was collected by three psychiatric nurses who had lots of experiences of working with people suffering from mental illness. Moreover, these data collectors had also prior experiences in conducting various psychiatric surveys. They received a 1 day theoretical and practical training on how to administer the data collection instruments, and how to approach patients, parents and guardians and obtain informed consent. In addition, the primary investigator, the psychiatrist, was assigned to organize, facilitate, and control the whole process of the data collection. His phone was always on for any consultation and queries. Informed consent was secured from patients or the guardians if the patients’ decision-making capacity was compromised and for those patients aged less than 18. Parents and guardians were required to leave the room until data collectors had completed interviewing patients to avoid response bias in the case of minors. No refusal was noted from the parents or guardians. After finishing the interview with minors, parents and guardians were interviewed for additional information with the consent of patients. Usual care treatments were provided by psychiatric nurses in consultation with psychiatrists. Moreover, patients were allowed appointments to visit psychiatric clinic for follow up and to get future psychiatric care plan.

### Data management

Every week, the principal investigator checked the completeness and consistency of the information in the questionnaires. Data was entered and analyzed using IBM SPSS statistics 21 software package. Descriptive statistics was used to present frequencies, means and standard deviation. Chi square test was used to present association. Crude odds ratio and 95% confidence interval were used to show the direction of association. Since our sample size was small we have not done multivariate analysis.

## Results

### Socio-demographic characteristics of the study participants

During the study period 101 individuals showed up in the emergency OPD with complications of suicidal attempt. Among these five of them died while they were on treatment at the emergency OPD. A total of 96 individuals were referred to the psychiatric clinic and all of them volunteered to be part of the study. Among the study participants 56 (58.3%) were females and 74 (77.1%) were from Hawassa city. The Mean (SD) age was 21.5 (8.0) years. Seventy-five (78.1%) of them were aged below 25 years. The majority, 91 (94.8%) were Christians, of whom 54 (59.3%) were Protestant while 37 (40.7%) were Orthodox Christians. Eighty-four (93.8%) participants attended grades 7 up to 12. Sixty-one (63.5%) participants were students by the time they attempted suicide. Eighty-one (84.4%) were single. The majority, 87 (90.6%), of the participants were living either with their parents or siblings. Sixty-eight (70.8%) of the participants received economic support from their parents or close relatives (Table 1).

**Table 1:** Socio-demographic characteristics of suicide attempters at general hospitals in Hawassa city from Nov 2014–Aug 2015

Characteristics	Number (%)
Age	
14–24	75 (78.1)
≥25	21 (21.9)
Sex	
Male	40 (41.7)
Female	56 (58.3)
Religion	
Protestant Christian	54 (56.2)
Orthodox Christian	37 (38.5)
Others	4 (4.1)
Place	
Hawassa city	74 (77.7)
Sidama zone	17 (17.7)
Others	5 (5.2)
Ethnicity	
Sidama	46 (47.7)
Wolayita	17 (17.7)
Amhara	15 (15.6)
Oromo	9 (9.4)
Others	9 (9.4)
Marital status	
Married	15 (15.6)
Single	81 (84.4)
Lives with	
Either parent or sibling	87 (90.6)
Spouse and children or a lone	9 (9.4)
Occupation	
Students	61 (63.5)
Self employed	16 (16.7)
House wife	9 (9.4)
Unemployed	6 (6.2)
Government employ	4 (4.1)
Income sources	
From parent or close relatives	68 (70.8)
Has personal income	28 (29.2)
Educational status	
Unable to read and write	3 (3.1)
Read and write	1 (1.0)
Primary school	6 (6.2)
Secondary school	84 (87.5)
Tertiary	2 (2.1)

### Methods of suicide attempt and clinical states at arrival and discharge

Forty-two (43.8%) of the study participants attempted suicide by ingesting pesticide poisons, 37 (38.5%) by ingesting a corrosive agent, and the remaining 17 (17.7%) used one of the following three methods: hanging, medication overdose or cutting their neck with a knife.

During arrival at the emergency services, 79 (78.2%) were alert, 11 (10.9%) were lethargic and 11 (10.9%) were in a comatose state. Among these, 5 (4.9%) of them died within few minutes of arrival at the emergency OPD while 9 (9.4%) of them were sent back to their homes within few minutes to hours of stay at emergency OPD. On the other hand, 50 (52.1%) of the study participants were made to stay in the hospital for around 12 h while the remaining 37 (38.5%) were kept in the hospital for more than 24 h either for observation or because of having serious medical complications. The maximum hospital stay was 72 h. Moreover, among the admitted patients, 83 (95.4%) of them were discharged from the hospital without having any suicide related complications while only 4 (4.6%) were discharged with non-serious complications such as scar marks, slurred speech and esophageal discomfort during swallowing.

The majority, 84 (87.5%) of the patients attempted suicide inside their living rooms while the rest in the field, in the forest, in the back yard and inside wash rooms. Fifty-five (57.3%) of the suicidal attempts were found and intervened by a parent while 21 (21.9) and 13 (13.5%) were terminated by a sibling, and by close friends, respectively. As an immediate intervention, 62 (64.6%) of the family members had given them milk whereas the rest did not get any intervention except being brought to the hospital (Table 2).

**Table 2:** Situation of suicide attempters at general hospitals in Hawassa city from Nov 2014–Aug 2015

Variable	Number (%)
Methods of suicidal attempt	
Hanging	8 (8.3)
Organophosphate poisoning	42 (43.8)
Medication overdose	6 (6.2)
Bleaching agent	37 (38.5)
Others	3 (3.1)
Immediate reason for suicide	
Quarrel with the family	59 (61.5)
Problem related with love affairs	6 (6.2)
Quarrel with spouse	3 (3.1)
Quarrel with friends	1 (1)
Academic difficulties	4 (4.2)
Financial crisis	6 (6.2)
Unplanned and unwanted pregnancy	10 (10.4)
Other	(7.3)
Clinical status during arrival^a^	
Lethargic	11 (10.9)
Comatose	11 (10.9)
Alert	79 (78.2)
Hospital stay	
Sent home immediately	9 (9.6)
Kept for ≤12 h	50 (52.1)
Kept for more than 24 h	37 (38.5)
Impulsivity of suicide attempt	
Within 5 min of decision	30 (31.3)
After 5 min of decision	66 (68.8)
Intention of suicidal attempt	
I made the attempt to kill myself and it was only luck that I did not succeed	32 (33.3)
My attempt was a cry for help and I did not intend to die	64 (66.7)
Attitude towards being survive	
Angry	9 (9.4)
Glad	85 (88.5)
Indifferent	2 (2.1)
Warning sign	
Yes	41 (42.7)
No	55 (57.3)
Type of warning sign	
Hopelessness	33 (34.4)
Threatening to hurt or kill self	4 (4.2)
Looking for ways to kill self	3 (3.1)
Talking or writing about death, dying or suicide	1 (1.0)
Recent(≤6 months) hospital visits	
Yes	76 (79.2)
No	15 (15.6)
Reason for hospital visit	
Medical	76 (95.0)
Psychiatric	4 (5.0)
Site of suicidal attempt	
Inside living room	84 (88.4)
Inside washroom	2 (2.1)
Backyard	1 (1.1)
Field	4 (4.2)
Forest	2 (2.1)
Others	1 (1.1)
Support for suicide failure	
Parent	55 (57.3)
Siblings	21 (21.9)
Friends	13 (13.5)
Close relative	1 (1.0)
Neighbor	5 (5.2)
Passer by	1 (1.0)
Psychiatry diagnosis	
Present	3 (3.1)
Absent	93 (96.9)

^a^The percentage was calculating among 101 patients presenting to emergency OPD while the percentage of other variables were calculated among 96 study participants included in the study

### Psychosocial factors and clinical situations before suicidal attempt

Quarreling with a family member was reported by 59 (61.5%) of the participants as the immediate reason to attempt suicide. Twelve (12.4%) of the study participants reported problems related to their love affairs and financial crises, and 8 (8.3%) were due to other reasons like quarreling with a spouse or friends, and academic difficulties. On the other hand, among female study participants, 10 (10.4%) of them attempted suicide because they had unplanned and unwanted pregnancy (Table 2). Though among 56 females study participants 10 of them attempt suicide because of having the unplanned and unwanted pregnancy, a correction has to make on table two variable said "Immediate reason for suicide". The figure for unplanned and unwanted pregnancy 10 (10.4)-(it is not 12 (12.5). This percentage is calculated out of 96 total study participants.

Nevertheless, 64 (66.7%) of the participants reported that their intention for attempting suicide was a cry for help while 32 (33.3%) of them said they made the attempt to kill themselves; however, it was only luck that they did not succeed. Eighty-five (88.5%) of the study participants were glad to survive from their suicidal attempts without serious complications while 9 (9.4%) felt angry since they did not succeed. Among the 9 patients who had felt angry, 8 of them reported that the intention of their attempt was wanting to truly kill themselves. Moreover, 41 (42.7%) of the study participants had shown warning signs to kill themselves before their action; a feeling of hopelessness was reported by 33 (80.5%) of them as a warning sign. Seventy-six (79.2%) individuals had history of hospital visits within 6 months prior to their suicidal attempt for different medical reasons. Only, 5 (6.2%) had visited the hospital for psychiatric reasons. But none of them were asked about suicidal ideation (Table 2).

### Prevalence of mental illness among suicide attempters

Mental illness was found only in three (3.1%) participants (Table 2). All of them were males. Among these, two of them were suffering from major depressive episodes and one participant had a history of alcohol, khat and cigarette abuse. Depressed mood nearly every day; loss of interest, decreased appetite, talking slowly, easy fatigability, feeling of worthlessness, difficulty in concentrating, and suicidal ideation were the symptoms reported by two of the patients with depression.

Thirty (31.2%) of the study participants attempted suicide within 5 min of their decision to kill themselves. Among these, 18 (60%) of them were male. Males were found three times more likely to attempt suicide impulsively than females (COR = 3, 95% CI 1.2–7.3) (Table 3).

**Table 3:** Cross-tabulation of gender vs. socio-demographic and clinical variables at general hospitals in Hawassa city from Nov 2014–Aug 2015

Variables	Gender		χ2 test, *P* value
	Male n (%)	Female n (%)	
Age			
14–24	29 (72.5)	46 (83.6)	1.73, 0.19
≥25	11 (27.5)	9 (16.4)	
Marital status			
Married	6 (15)	9 (16)	0.02, 0.89
Single	34 (85)	47 (84)	
Income sources			
From parent or close relatives	25 (62.5)	43 (76.8)	2.3, 0.13
Has personal income	15 (37.5)	13 (23.2)	
Warning sign			
Yes	14 (35)	27 (48.2)	1.67, 0.20
No	26 (65)	29 (51.8)	
Intention of suicidal attempt			
I made the attempt to kill myself and it was only luck that I did not succeed	10 (25)	22 (39.3)	2.14, 0.14
My attempt was a cry for help and I did not intend to die	30 (75)	34 (60.7)	
Impulsivity of suicide attempt^a^			
Within 5 min of decision	18 (45)	12 (21.4)	6.03, 0.01
After 5 min of decision	22 (55)	44 (78.6)	

^a^COR of male gender = 3.0, 95% CI  1.2–7.3

## Discussion

We have evaluated a total of 96 patients. Seventy-five (78.1%) of our study participants were below the age of 25 years. This finding is comparable with other studies which reported that the productive younger age groups are highly vulnerable to suicidal attempt [20, 24, 25, 44]. In terms of gender differences, female suicide attempters outnumbered male attempters with a ratio of 1.4. Consistent with our findings, female sex preponderance have been documented in previous studies [24, 25, 36, 45]. In general, male die three to four times more frequently by suicide than females [46, 47]; whereas, females attempt suicide two to four times more often than males [48, 49]. This discrepancy arises because of the method they use to kill themselves; males use more lethal methods such as hanging and firearm, while females use less lethal methods such as drowning or poisoning [47, 50]. Despite the high prevalence of suicidal attempt in females, there is lack of attention to female suicidal behaviors resulting from viewing female suicidal behaviors as manipulative and non-dangerous [47, 51]. However, many studies in the past have shown that the risk of suicidal attempt increases with history of previous suicidal attempt; 10% of the individuals re-attempt suicide within 6 months while 42% re-attempt suicide within 21 months of follow up periods [52, 53]. Moreover, among the risks of suicide, suicidal attempt was said to be the best predictor of suicide; for example, succeeding in suicidal act is 30–40 times higher in those individuals who attempted suicide than the general population [19].

The most striking finding in this study was the low prevalence rate of mental illness among study participants; appearing only in 3 (3.1%) of the study participants. This is in contrast to other studies [21–23] where mental illness was found highly prevalent among individuals who attempted suicide; in the range of 60–80%. The presence of mental illness among individuals who attempted suicide has also been evidenced by many other studies. For example, a study conducted on 114 suicide attempters at a general hospital in Helsinki had shown that prevalence of mental illness may reach as high as 98% among suicide attempters [54]. Among mental disorders reported in patients with suicidal behavior, depression was the commonest one. A study conducted by Suominen et al. [54] in 1996 had reported that 67% of patients who attempt suicide had been suffering from depression. Furthermore, a study conducted by Kumar et al. [55] in 2006 showed that 81% of their study participants were suffering from one form of mental disorder; depression being the commonest one. Even if further study will be needed to find why mental illness is low among individuals who attempted suicide in our settings, being in a younger age, before the expected age of developing common mental disorders, could be one of the possible explanations why mental disorders were low in this study. Although MINI PLUS was said to be a highly reliable and valid instrument to pick major mental illnesses [43, 56], and in spite of its use in various countries, including Sub-Saharan African countries [37–39], the absence of its validation in our setting could have its own contribution to the low prevalence of mental illness in this study. Moreover, except for antisocial personality the instrument does not incorporate other personality disorders such us borderline personality disorders. Given the high prevalence of suicide attempt risk, as high as 70% of patients with borderline personality disorders [57, 58], the lack of its inclusion in the MINI PLUS may also have its own role to lessen prevalence of mental disorders in this study.

The other important finding of this study was the impulsive nature of the study participants; 30 (31.2%) of them attempted suicide impulsively. This figure is higher than the study conducted by Simon et al. [31] in 2001, but lower than that of Williams et al. [59] in 1980; 24 and 40%, respectively. In this study impulsivity was found associated with male gender (COR = 3, 95% CI 1.2–7.3). Male gender association with impulsivity was also reported by previous studies [31, 59].

Like other studies [60–63], ingesting poisonous materials was found to be the most commonly used method to attempt suicide; 79 (82.3%) of the study participants ingested either pesticide poisons or corrosive agents. In contrast to these studies, hanging was found the least method used by the victims in this study, that is by only 8 (8.3%) of the study participants. Consistent with other studies [64–66], females predominantly use either pesticides or corrosive agents than male. Easy availability of lethal methods at home or around may play an important role in the methods of choice, particularly if the nature of suicide attempt is an impulsive one [67]. In this study, a significant number of participants used corrosive agent, medication overdose and pesticides which are used for domestic purposes in a majority of Ethiopian households. Despite some controversy in their effectiveness in reducing suicide attempts, restricting the availability of lethal methods is generally helpful to prevent deaths related to suicide [68, 69].

Though many studies [24, 25] suggest that having previous suicidal attempts were the main risk factors for next suicidal attempt or suicide, in this study, no participant had a history of previous suicidal attempt. This might be explained by the age of participants in this study. Since the majority, 75 (78.1%) are below the age of 25 years; the current suicidal attempt might be their first experience. The majority of our study participants reported psychosocial stressors as immediate reasons to attempt suicide. For example, 59 (61.5%) of the study participants had quarreled with a family member; 6 (6.2%) had problems related to their love affairs; 6 (6.2%) faced financial crisis. Moreover, among the female study participants, 10 (17.9%) of them attempted suicide because of unplanned and unwanted pregnancy. These finding show that our youngsters might have problems in solving psychosocial crises. Likewise, other previous studies conducted in India and other western countries, have reported that different life events to be important risk factors for deliberate self-harm/suicide attempt [23, 70, 71]. Similarly, an earlier study conducted by Siwach and Gupata reported that marital disharmony, economic hardships, and scolding/disagreement with other family members as the major precipitating factors [72].

The absence of skills to solve psychosocial crises and the presence of unsafe sexual practices among youths warn us to design strategies to prevent suicide. An urgent need to prompt education regarding a variety of family planning methods, and training on problems solving skills to younger generation to allow them detect problems early and get treatment timely might be necessary.

The study results may not be generalized since our sample size was small and derived from patients referred from emergency and inpatient services of only two hospitals in SNNPR. On the other hand, because of the stigma associated with suicide, families may not report the case to health professionals. Moreover, those patients who did not appear at the hospital because of non-serious suicidal attempt or died at home and after arrived at emergency department were automatically excluded from our study. Finally, not using a validating instrument and the absence of borderline personality disorder in the MINI PLUS have to be taken into consideration during the interpretation of the results of this study.

## Conclusions

The majority of the suicide attempters were below the age of 25. The prevalence of mental illness was found very low compared to those reported figures elsewhere, which are in the range of 60–80%. The presence of impulsivity among youngsters suggests that there is a need to develop suicide prevention strategies. Restricting lethal methods, like pesticides and corrosive agents, may either prevent or decrease suicidal attempts. Having a clear policy on how one can sale and possess these lethal agents is necessary. High numbers of the study participants reported various stressful life events as immediate reasons for suicidal attempt. These findings may suggest the need for the preparation of training programs on problem solving skills for this group of the population. In addition, public education on healthy coping mechanisms under stress and improving communication skills of youths are also crucial to prevent suicidal attempt. Our findings were exploratory, which signaled the need for further focused research to confirm these observations and systematically examine the relationship between the presence of mental illness and suicidal attempt with a larger sample size and more robust methodology.

## Abbreviations

MINI PLUs: Mini International Neuropsychiatric Interview version 5
SNNPR: Southern Nations, Nationalities and Peoples’ Region
OPD: outpatient department
